# GENOMEMASKER package for designing unique genomic PCR primers

**DOI:** 10.1186/1471-2105-7-172

**Published:** 2006-03-27

**Authors:** Reidar Andreson, Eric Reppo, Lauris Kaplinski, Maido Remm

**Affiliations:** 1 Department of Bioinformatics, University of Tartu, Tartu, Estonia; 2 BioData Ltd., Tartu, Estonia; 3 Estonian Biocentre, Tartu, Estonia

## Abstract

**Background:**

The design of oligonucleotides and PCR primers for studying large genomes is complicated by the redundancy of sequences. The eukaryotic genomes are particularly difficult to study due to abundant repeats. The speed of most existing primer evaluation programs is not sufficient for large-scale experiments.

**Results:**

In order to improve the efficiency and success rate of automatic primer/oligo design, we created a novel method which allows rapid masking of repeats in large sequence files, for example in eukaryotic genomes. It also allows the detection of all alternative binding sites of PCR primers and the prediction of PCR products. The new method was implemented in a collection of efficient programs, the GENOMEMASKER package. The performance of the programs was compared to other similar programs. We also modified the PRIMER3 program, to be able to design primers from lowercase-masked sequences.

**Conclusion:**

The GENOMEMASKER package is able to mask the entire human genome for non-unique primers within 6 hours and find locations of all binding sites for 10 000 designed primer pairs within 10 minutes. Additionally, it predicts all alternative PCR products from large genomes for given primer pairs.

## Background

Microarrays and other genomic technologies allow the testing of thousands of genomic regions from each sample. Most of these methods require PCR amplification to achieve sufficiently strong signals. Therefore, there is a growing need for automatic oligo design and PCR primer design methods. There is always a certain chance that the selected PCR primers have alternative binding sites on the template DNA. It is expected that numerous alternative binding sites of the PCR primers may cause failures in PCR. However, the exact relationship between the number of binding sites in the genome and the success rate is not known. The computational prediction of these unwanted interactions would help to increase the quality of microarrays and genomic PCR and to reduce the cost of related experiments.

A large variety of PCR primer design programs exist. Nevertheless, only few of them allow some kind of testing for primer uniqueness and/or testing for alternative products from the same template. Some programs use repeat libraries to avoid primer design from repeated regions. For example, a program called PC-RARE uses 8-mer frequency disparity at the 3' end of primers to avoid the risk of non-specific binding of primers [1]. FAST-PCR program allows homology search against the custom list of repeated sequences [2]. OLIGO6 [3] uses libraries that contain all the frequent 6-mer to exclude the primers that contain highly repeated motifs. A well-known PRIMER3 [4] can be used with the collection of repeats (repeat library) to avoid non-specific binding of primers. However, the primers are compared to a repeat library using the Smith-Waterman algorithm [5], which makes use of large libraries for the design of a large number of primers slow and therefore unpractical.

Some programs use suffix trees or suffix arrays to ensure the uniqueness of selected oligos. PICKY is an oligo design software that allows one to create unique hybridisation oligos [6]. This program uses a suffix-tree based algorithm to test the uniqueness of oligo candidates in a user-given sequence set. Rahmann presents a method that uses the longest common substring as a specificity measure for candidate oligos [7]. This algorithm is based on a suffix array with additional information that is efficient both in terms of memory usage and running time to rank all candidate oligos according to their specificity.

The masking of repeats on the template DNA is another common approach that is used to avoid non-specific binding. DUST [8] and TandemRepeatsFinder [9] are commonly used for masking simple (short) repeat motifs. RepeatMasker is a universal program that is used for masking out several kinds of repeats and therefore mostly used for this kind of sequence analysis [10]. Similarly, BLAST [11] can be used to mask the non-unique regions of the genome [12,13]. Onodera and Melcher have found that unfavored and preferred 3' end triplets existed in different viral genome sequences [14]. They recommend a scoring system incorporating empirical frequencies of different 3' end triplets and that information may be used in masking primer candidates with poor success rate in other genomic sequences.

Another widely used method related to the success of the PCR is prediction of all PCR products from the genome sequence. This process is known as electronic PCR (e-PCR) or virtual PCR (vPCR). Electronic PCR was first developed to detect the presence of sequence tagged sites (STS) in template DNA [15]. Virtual PCR is a web based service that predicts PCR products from different genomes [16]. SPCR is a recently published program that can help the user to choose a PCR primer pair giving the least possible non-targeted products [17]. Its algorithm is based on the hypothesis that the annealing of a primer to a template is an information transfer process. We will use the term 'e-PCR' for all similar computational predictions of PCR products throughout this paper. The process of e-PCR is typically performed with the help of sequence alignment software, which counts the number of identical or nearly identical matches between the primer and the template DNA. The BLAST program is most frequently used for this purpose in multiple applications [18-20]. Nevertheless, the speed of BLAST is not sufficient for e-PCR in large eukaryotic genomes with large number of primers. High-speed methods applicable to large-scale problems are becoming more important with the increasing number of genome sequences. The speed can be increased by using MEGABLAST [21], BLAT [22] or SSAHA [23] which are specifically designed for large scale sequence search and alignment. A primer design program called MuPlex includes BLAT software to align primer candidates against genomic DNA [24]. These methods are relatively fast, but unfortunately all of them require specific parsers to count all primer binding sites and to find all PCR products on the template DNA. PRIMEX [25], on the other hand is specifically designed for testing oligos and counting primer binding sites from genomic DNA. Another recently reported program, me-PCR [26], is designed for the detection of locations of STS markers in the human genome and is helpful for the detection of PCR products of any type. There is a similar program called In-Situ PCR (isPCR) [27] created by Jim Kent that is also designed for predicting possible PCR products that two primers could produce.

In this paper, we describe a novel and efficient method, which masks large sequence files for repeats, performs a rapid prediction of all binding sites of PCR primers and predicts possible PCR products. The efficiency of our program is compared to several existing methods.

## Implementation

### Components of the software package

The GENOMEMASKER package can be divided into two separate parts: 1. A repeat masking application (GenomeMasker with auxiliary programs), 2. An e-PCR application for predicting primer binding sites and PCR products (GenomeTester with auxiliary programs). They contain the following executables:

#### 1. GenomeMasker application

*glistmaker *– creates a so called blacklist of over-represented (occurring more frequently than user-defined threshold) primer binding sites in a given genome.

*gmasker *– performs a binary search in the blacklist for each studied FastA sequence and masks the words present in the blacklist.

*gm_primer3 *– a modified PRIMER3 program that is able to use *gmasker *output for primer design.

#### 2. GenomeTester application

*gindexer *– creates binary index files containing locations of all the predicted binding sites in a given genome.

*gtester *– performs a binary search in index files for each primer to locate all their binding sites in a given genome.

*gt2multiplex *– extracts nucleotide sequences of all PCR products from template sequence using the output information of *gtester*.

### The GenomeMasker algorithm

The first part of the package – GenomeMasker application – contains programs required for the masking of repeated primer binding sites on the template DNA. The program *glistmaker *reads through the template sequence(s) and counts the number of occurrences of each word of user-defined length. After that it creates a blacklist containing only over-represented words, encoded into 32 bit integers. The encoding is done by allocating two consecutive bits for each nucleotide in a word. Thus the maximum word length in current implementation is 16 nucleotides. We define the over-represented word as the sequence that occurs in the given genome more times than a user-given cutoff (eg. 1, 2, 3, etc.). The entire list of encoded words is sorted for faster access. The input for *glistmaker *is one or more FastA files (either DNA template or genome/chromosome sequences).

The program *gmasker *uses blacklist file as a reference to mask the sequence file in FastA format. It iterates over the whole input sequence with step 1 nucleotide and checks forward word, its reverse complement or both against the blacklist. If a given word is in the blacklist, the corresponding word in the template sequence is masked. Only one nucleotide at the 3' end of the matching word is masked by default, because this should be sufficient to avoid most of the low-success PCR primers. Nevertheless, the user can define how many nucleotides will be masked by *gmasker *with a special option. The output of the program is a FastA file identical to input file, except that it contains masked nucleotides (uppercase letter changed to lowercase letter) in regions where primers should not be selected. Instead of uppercase-lowercase masking, any user-defined character can also be used for masking by *gmasker*. However, the lowercase masking is recommended because it maintains the sequence information at masked sites and allows subsequent primer design from the masked sequence.

The program *gm_primer3 *is a modification of PRIMER3 that is able to use the lowercase-masked sequence for primer design. The overall functionality and algorithm of the program is the same as in the original PRIMER3, but we have added a new filtering feature that rejects the primer candidates with lowercase letters in 3' end. However, *gm_primer3 *can be used to design primers from templates masked by other methods as well.

### The GenomeTester algorithm

The second part of the package, the GenomeTester application, contains programs for predicting primer binding sites and PCR products in long template DNA sequences, e.g. eukaryotic genomes. The program *gindexer *is needed to create index files for *gtester *to work. It creates 4 different index files for each sequence file – all words starting with A, C, T and G nucleotide. For example, four index files will be created for 24 human chromosomes creating a total number of 96 files. The creation of a separate index file for each nucleotide is necessary to reduce the file size and memory usage of *gtester*. In an index file we keep 8 bytes of data for each occurrence of each word in the whole genomic sequence. The first 4 bytes represent the word, encoded as 32 bit integer, 2 bits per nucleotide. The last 4 bytes contain the position of the current occurrence of the word in the genomic sequence. The word length can be specified by the user within the range between 8 and 16 nucleotides. The entire file is sorted by the encoded word (by first 4 bytes) in order to allow high speed binary search with the *gtester*. The sequence files of chromosomes in FastA format are used as input of *gindexer*.

The program *gtester *works in four steps. During the first step, it creates an array structure of primer pairs. For each primer pair, it takes a word of given length (the same length as was used for creation of index files) from the 3' end of the primer and creates 2 words – one original and one reverse complement. In step 2, a binary search is performed with all words in the array against index files. Another list with the locations of the binding sites is created in step 3. Finally, the program finds all PCR products that are possibly synthesized by the given primer pair hybridizing on both sense and antisense chains of the DNA. PCR products generated by a single primer are also considered. For both parts of the package, primer binding sites can be modelled with a custom, user-defined word length.

### Advantages of our implementation

The speed of the programs in this package is achieved by extensive pre-processing of the genomic data. Both applications, GenomeMasker and GenomeTester, require preprocessed files to work efficiently. During the creation of these files all the locations in the genome are counted, sorted and recorded in a binary format. The blacklist of GenomeMasker contains the list of all over-represented binding sites. The index files of GenomeTester contain a list of all binding sites together with their location in the genome. There are two reasons why GenomeTester and GenomeMasker are faster than most other similar applications. First, both of them use the fast binary search algorithm which runs in O(log *n*) time. The binary search algorithm [28] can be briefly explained as follows. The search itself begins by examining the value in the center of the list (index). These values in the list are sorted, so the program knows whether the value occurs before or after the center value. Then the program searches through the correct half in the same way as before. Those cycles will be repeated until the searched value is found or until there are no more values to check. The second advantage of our programs is an on-demand memory-mapping technique that allows us to achieve optimal speed for analyzing both small and large input datasets. The programs *gmasker *and *gtester *also use an on-demand memory-mapping technique. Instead of pre-reading the entire index into memory they only read the requested parts (positions in the middle of intervals). Those parts will stay in a memory cache and thus the search speed per string will increase gradually when using more search strings, as more of the index will need to be read into the memory.

### Parameters for comparing different programs

All software programs were tested on assembled chromosome sequences derived from ENSEMBL database Human 19.34 (NCBI Build 34). The computational performance tests described here were obtained by running all the programs on a 2.66 GHz Intel Xeon™ processor machine with 6 GB of RAM.

RepeatMasker (version 2004/03/06) was used with different sensitivity parameters -s, -q and -qq. -s means "slow search", which is 0–5% more sensitive and 2–3 times slower than default. -q is a "quick search", 5–10% less sensitive and 2–5 times faster than default. -qq is a "rush job", about 10% less sensitive and 4–>10 times faster than default. We used RepBase Update [29] 8.12 library (6 March 2004) of repeated motifs in human genome. DUST was used with default parameters. TandemRepeatFinder was used with alignment parameters (match, mismatch, indels) 2, 7 and 7, minimum alignment score to report repeat 50 and maximum period size 500. GenomeMasker blacklist was created with word length 12 and word length 16 and with over-represented cutoffs 1000 and 10 respectively. The masking program *gmasker *was used with masking letter parameter 'l' (lower-case masking) and masking type parameter 'target 500 501'.

The SSAHA indexes were created with the word length parameter (-wl) 10 and step length (-sl) 1. SSAHA searches were performed with match lengths (-ml) 16. PRIMEX indexes were created with default parameters and word length (-wl) 10. The number of mismatches allowed in each lookup table word (-m1) and the number of mismatches allowed in the entire query (-m2) were set to 0 and 1 respectively. Me-PCR was executed with parameters: word size (W) 11, number of mismatches allowed (N) 0, margin (M) 500 and default PCR size (Z) 240. The parameters as word size (-W) 12 and maximal total length of queries for a single search (-M) 16 were used for MEGABLAST. All results were parsed with word length 16 (32 bits for BLAST and MEGABLAST). The maximum size of the PCR product (-maxSize) was set to 1000 bp with stand-alone isPCR. GenomeTester indexes were created with word length 16 and the program *gtester *was used with default parameters.

## Results

### The GenomeMasker package

Predicting the number and location of PCR primer binding sites in large genomes can pose computational challenges. Therefore we have designed a software package GENOMEMASKER that helps to achieve a fully automatic sequence masking and PCR primer testing for large genomic applications. The first part of the package – GenomeMasker – is designed to mask all repeated primer binding sites in the template sequence to avoid selection of such primers. The essence of sequence masking is in finding and marking sequence regions with specific properties, e.g. repeated regions of sequence. Masked nucleotides are typically replaced by character 'X' or converted to a lowercase character. Unlike the widely used masking program RepeatMasker, GenomeMasker masks only the 3'-terminal nucleotide of each repeated word, which is sufficient to avoid primer design from the repeated region. To be able to use that masked sequence for designing unique PCR primers, the PRIMER3 program was modified to distinguish between upper- and lowercase letters in a masked template file. If a primer candidate ends with a lower-case letter it will be rejected by PRIMER3 and unique primer will be designed from the remaining candidates. However, other primer design methods that can use masked sequence could also work with the GenomeMasker output file.

Another important test for genomic PCR applications is the prediction of the number of all possible PCR products, which a given primer pair can generate from a given genome. This can effectively be done by the second part of our package called GenomeTester. GenomeTester counts and locates all potential binding sites of the PCR primer pair in the genome and predicts the location of all PCR products that could be generated with these primers. These two main methods – GenomeMasker and GenomeTester – can be used independently.

Our software models the PCR primer binding site as 100% identical match to a continuous string (word) from primer's 3' end with a fixed length. User defined length in the range of 8 and 16 nucleotides can be used. The bindings with mismatches are not modeled for several reasons. Firstly, for each 16-mer oligonucleotide there are 48 variants with a single mismatch. Counting the number of occurences and keeping track of genomic location of all these variants would increase the computing time significantly. Secondly, it is not known whether counting mismatch containing primer binding sites helps to predict the PCR success rate better than counting full-match primer binding sites. Until the relationship between the number of mismatched binding sites and PCR success is known we prefer not to use mismatched binding sites.

### Workflow of the program package

**Figure 1:**
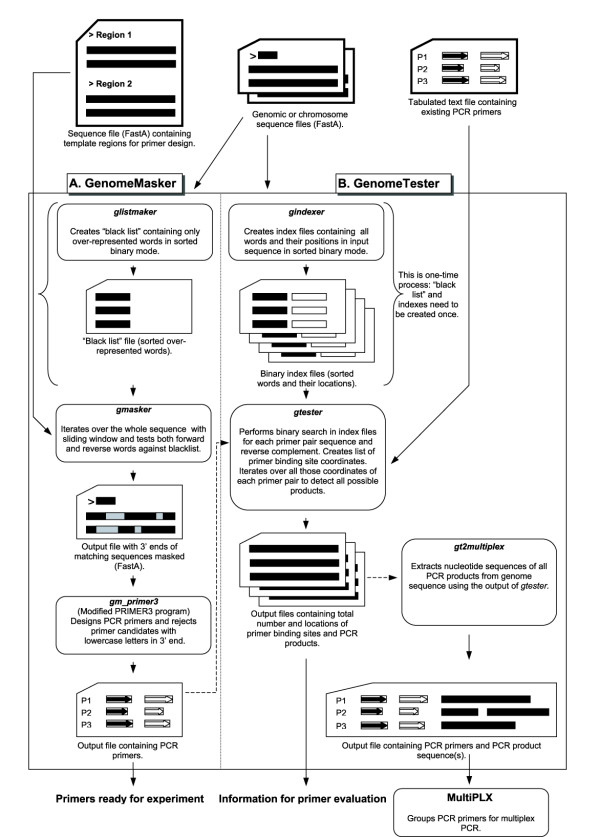
**The workflow of the two separate units in GENOMEMASKER package: GenomeMasker (A) and GenomeTester (B)**. Boxes with rectangular corners describe data structures, rounded boxes describe procedures performed by different programs and boxes with bold outlines denote the main input files. Procedures indicated by dashed lines are optional.

Figure 1 shows the basic workflow of GENOMEMASKER package. In the initial step the blacklist file for GenomeMasker and the binary index files for GenomeTester have to be created by the user from the genomic data in a FastA format. These procedures have to be performed only once for a given set of genomic data and chosen word length. After creating the blacklist file, the user can start masking sequence files containing template DNA regions (in FastA format) with the *gmasker *program and design unique PCR primers with the *gm_primer3 *program. If the user already has PCR primers (tabulated text file), GenomeTester can evaluate the primer pairs by counting the number of all binding sites and possible PCR products and by recording their coordinates. Additionally, with GenomeTester the user can create a special file containing primer and product sequences, which can be used for multiplexing PCR primers into compatible groups by using the previously published program MultiPLX [30].

The creation of either a blacklist for GenomeMasker or the indexes for GenomeTester from the entire human genome takes approximately three hours on a Linux server with 2.4 GHz Xeon processor, at least 2 GB of RAM and SCSI disks. Once the preprocessing is done, the masking, primer design and primer testing steps are extremely fast. The algorithmic details are more thoroughly described in the chapter "Implementation and methods".

### GenomeMasker: comparison of repeat masking methods

**Figure 2:**
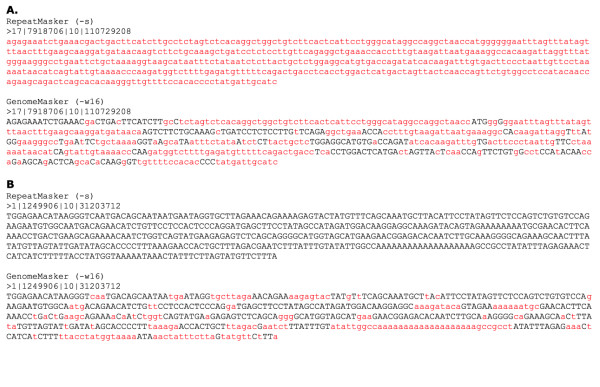
**Examples of masking style of different masking programs**. Masked nucleotides are shown by red lowercase letters. There are some DNA regions that are extensively masked by RepeatMasker (A) and some regions where RepeatMasker does not find any repetitive motifs (B). Both programs are executed at similar sensitivity level.

To compare the properties of our package with the properties of other repeat detection and masking methods we chose 1000 random regions from the human genome, each 1000 bp long. The sequences were masked with RepeatMasker and GenomeMasker. PCR primer design was attempted from each sequence. Both masking programs were used at similar sensitivities – the overall fraction of masked nucleotides was similar (41% and 37% respectively). The results of masking are shown in Figure 2. The general tendency is that masking by GenomeMasker is more detailed than masking by RepeatMasker. In sequences masked by RepeatMasker short repeats are often not detected. Incomplete RepBase libraries may be one of the causes of that. On the other hand some other DNA regions are extensively masked by RepeatMasker and cannot be used for primer design. GenomeMasker, however, masks only a single nucleotide in the 3'-end of each over-represented word it finds. This creates a more detailed masking pattern and allows the design of primers inside complicated regions between repetitive sequences. Detailed masking is legitimate because GenomeMasker assures that any non-masked word is not repeated in the genomic DNA sequence.

We compared the properties of primers designed from template DNA masked with different programs (Table 1). In addition to GenomeMasker and RepeatMasker we studied the primers designed from the non-masked templates using the repeat detection library built-in into the PRIMER3 program. We also used short repeats masking programs DUST and TandemRepeatsFinder. The amount of outcoming primers and their properties are rather different. It turns out that using only the default repeat library of PRIMER3, masking with DUST or TandemRepeatsFinder is not sufficient to design non-repeated primers. As much as 39% of primers designed using PRIMER3 repeat library still occur in more than 10 locations in the genome, which, according to our estimates can reduce the success rate of PCR. RepeatMasker is good in avoiding such repeated regions, but unfortunately it tends to mask large regions thus making the primer design impossible in complicated regions. Primer design was not possible in around 31% of randomly chosen regions masked by the RepeatMasker.

**Figure 3:**
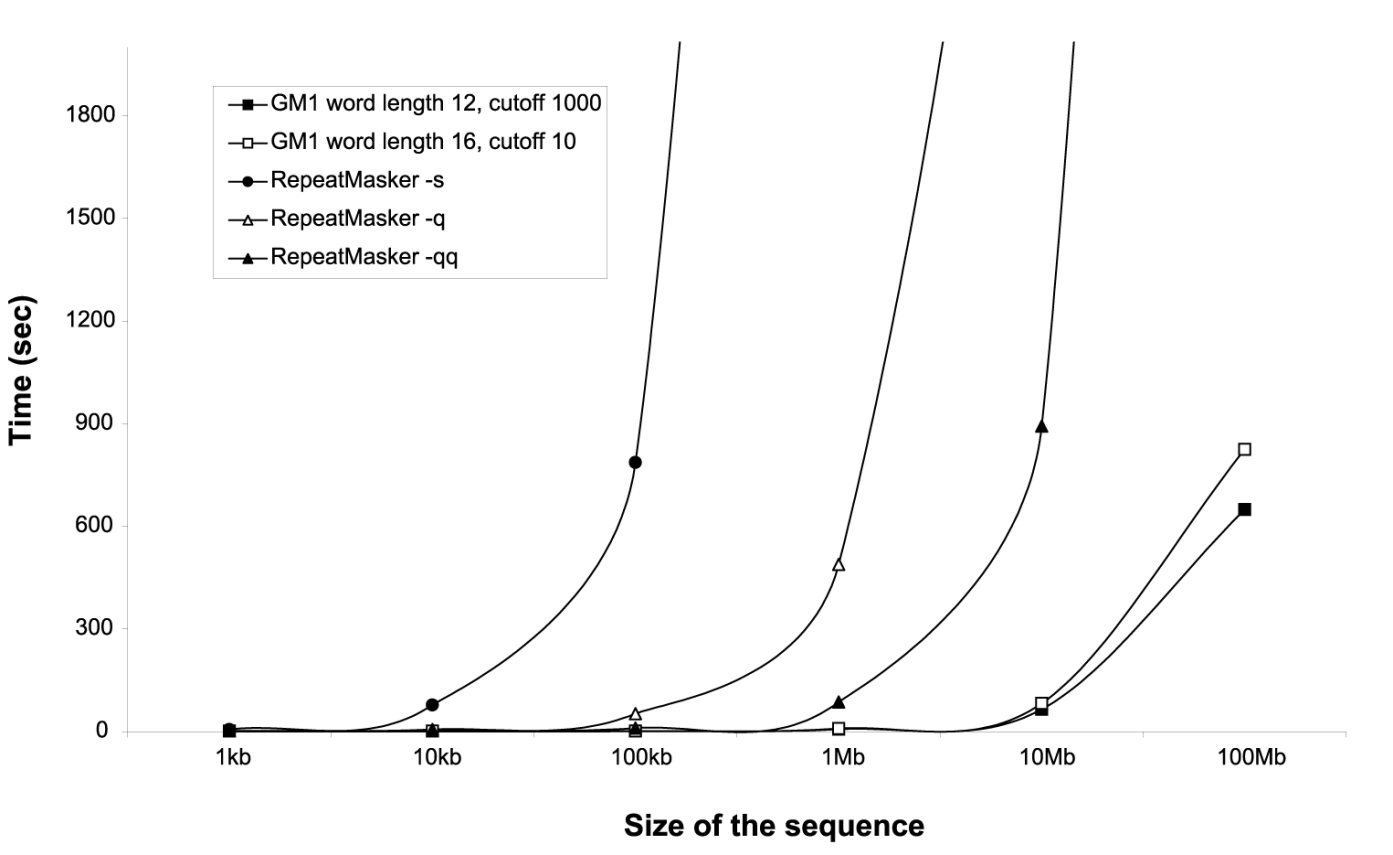
**The speed test between two masking methods: RepeatMasker and GenomeMasker**. Both programs were tested with several parameters and different template sizes. Masking of the entire human genome takes 6–7 hours with the GenomeMasker. Programs were executed on a 2.66 GHz Intel Xeon™ processor with 6 GB of RAM.

The computational performance of GenomeMasker compared to RepeatMasker is shown in Figure 3. RepeatMasker was used with several different sensitivity parameters and GenomeMasker with two different word lengths. GenomeMasker is at least 10 times faster compared to RepeatMasker. To simplify the primer design process, the user can pre-mask the entire human genome within 6–7 hours and subsequently use the masked genomic sequence for various primer design tasks.

### GenomeTester: comparison between e-PCR methods

Although the masking of template sequences with GenomeMasker avoids low-quality primer design, some primer pairs may still produce two or more alternative PCR products which should be avoided. Also, users may have existing primer pairs that they would like to evaluate against the given genome for the number of binding sites. Therefore, it may be necessary to perform a search against the entire genome for primer binding sites and detect the location of possible products. Such searches are typically done with sequence homology programs (eg. BLAST) or with dedicated e-PCR programs (eg. me-PCR). The computation time and the memory requirements of e-PCR programs are important factors, particularly when dealing with large datasets like eukaryotic genomes. To compare the speed of different methods we created five datasets, consisting of 1, 10, 100, 1000 and 10000 randomly selected primer pairs. All datasets were subjected to the e-PCR against the human genome with seven different programs and the computing time of the results was recorded.

**Figure 4:**
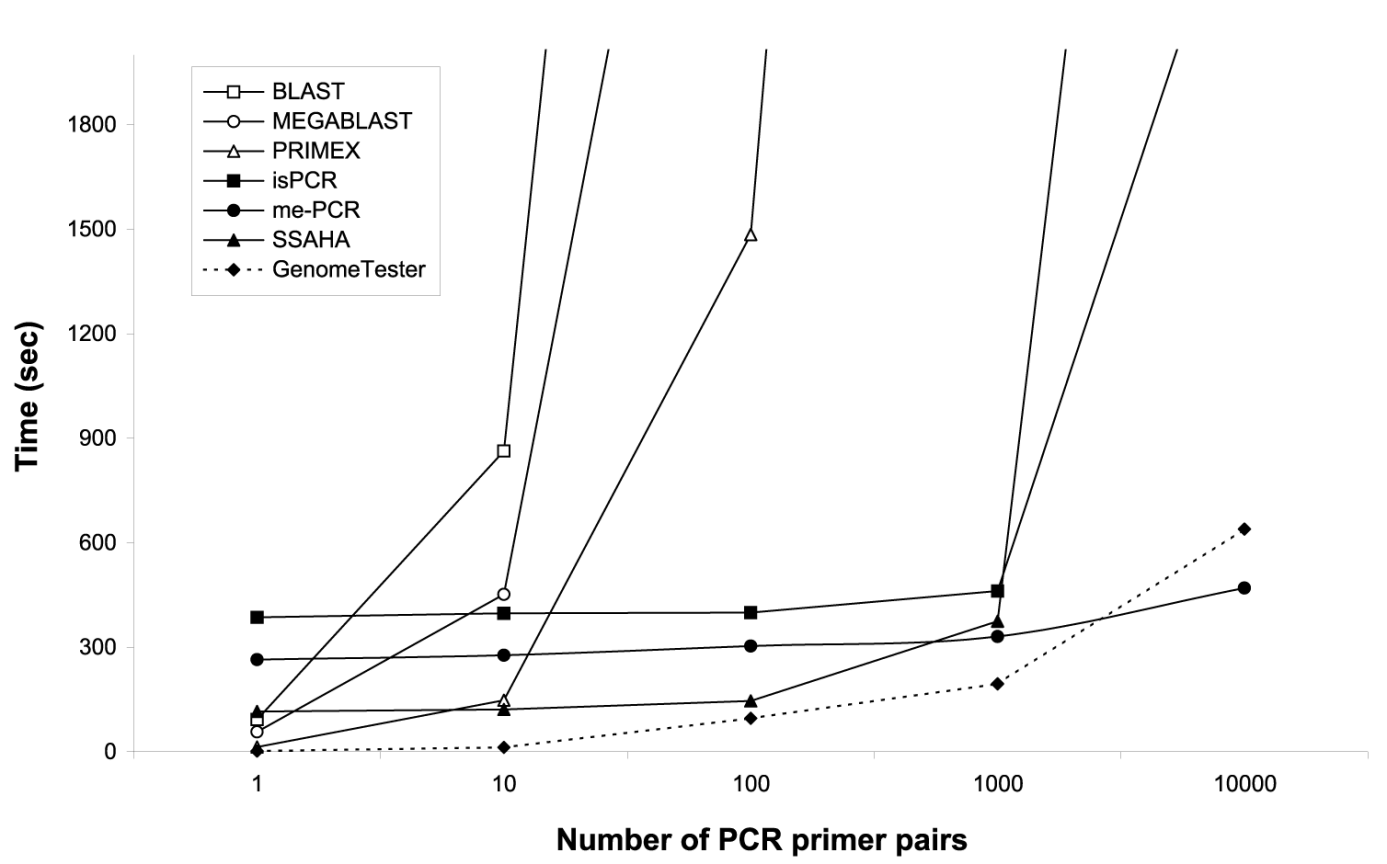
**The performance test between various alignment and e-PCR methods**. We compared the speed of seven different methods with five datasets, consisting of 1, 10, 100, 1000 and 10000 randomly selected primer pairs. All datasets were subjected to the e-PCR against the human genome with different programs and the computing time was recorded. All programs were executed on a 2.66 GHz Intel Xeon™ processor with 6 GB of RAM.

The results of the comparison are shown in Figure 4. The speed difference between the fastest methods and more traditional sequence homology search programs like BLAST and MEGABLAST is more than 100-fold. me-PCR program seems to be more effective for larger datasets than other e-PCR methods. However, one should keep in mind that although the recent e-PCR methods like me-PCR and isPCR are very fast, they are designed to locate PCR products only. Other studied programs are able to record the location and number of all primer binding sites.

Physical memory requirement for the e-PCR procedure on human chromosomes is approximately 1GB for SSAHA, 500 MB for GenomeTester and isPCR and ca 300 MB for other methods. Please note that all the programs shown in Figure 4 were compared in standalone mode. The performance of most of the programs can be increased by using server-client architecture (with all the genomic data stored in server RAM).

## Discussion

Large-scale genomic studies often require amplification of genomic DNA by PCR. Therefore, automatic PCR primer design is one of the critical steps in such studies. We have created a novel method for masking repeated regions in sequences. It allows masking of the entire template DNA before primer design to avoid the consideration of poor primer candidates. GenomeMasker is able to identify and mask repeating words that have not been included in the current repeat libraries. This, combined with a specific 3'-end masking technique, allowed us to achieve a more sensitive masking than the existing approaches. Programs like DUST and TandemRepeatsFinder are designed for masking short repeat motifs and are faster than our method, but their ability to find different types of longer repeats is lower, as demonstrated in Table 1. Masking repeats may help to increase PCR success rate, but this might not be sufficient for some applications. For example, genotyping [31] requires that a unique PCR product must be generated from the human genome. Therefore, additional e-PCR step is still required after candidate primer pair is selected, which in our case is done with the GenomeTester application. The method presented in this paper offers the possibility to increase specificity and speed of finding non-unique words by using word indexing and binary search algorithm compared to other repeat-masking and e-PCR methods. The package also contains an optional primer design program that can be replaced with desired software. Therefore, the main advantage of our method is speed, unique masking style and the possibility to quickly locate alternative PCR primer binding sites and products.

All programs in our test must read the genome sequence information from a hard drive. This information is typically pre-processed and saved into a database or an index in a specific binary format. Databases or indexes that need to be read from hard drive are rather large. Total size of indexes for the human chromosomes is approximately 1 GB for BLAST and MEGABLAST, 11 GB for SSAHA and 22 GB for GenomeTester. When executed, most programs initially read all database/index data into RAM, which speeds up the search, particularly for large datasets. However, for searching only a few primer pairs the entire database/index may not be necessary. The pre-processed and sorted index of GenomeTester allows identifying locations of the given primer pair by reading only a fraction of the index file.

Our package is based on finding the exact matches between sequences. Newer methods [25] and algorithms [32] allow considering mismatches when comparing oligos against large genomic sequence. Whereas it would be possible to enhance our programs so that the bindings with single mismatches would be taken into account, the current implementation would require about 45–48 times more computing time for both *gtester *and *gmasker*. The number comes from the fact that there are 3 mismatched variants per each nucleotide within a given word. For example, 16-mer oligonucleotide would have 48 different single-mismatch oligos. We could forbid the mismatch at 3'-end of oligo, but nevertheless the number of different variants to search is huge. The problem is particularly serious with the GenomeTester which keeps track of the location of each potential binding site. Thus, the memory requirement for storing all the locations would be enormous. Also, there is no good model to estimate how different mismatches should be weighted. For example, it is usually expected that mismatches near the 3' end disturb primer binding and subsequent replication matter much more than the ones near 5' end. The exact dependence between mismatch location and binding strength is not known. Additionally, the strength of the mismatched DNA duplex is somewhat weaker than the duplex with exact matches [33]. Thus, the relative influence of mismatched binding sites to the success rate of PCR compared to fully complementary binding sites is poorly understood and their importance in predicting PCR success cannot be estimated without extensive experimental studies.

However, we have examined the correlation between the number of perfect match binding sites and the number of binding sites with single mismatch. We have taken all strings with length 12 nucleotides from the human genome. For each oligonucleotide, two things were counted 1) the number of binding sites in the genome as 100% identical match and 2) the number of binding sites in the genome containing single mismatch. These two numbers were plotted against each other and a strong linear correlation was observed (with correlation coefficient r = 0.938). As these two values are strongly correlated we would expect them to be almost equally efficient predictors of PCR success. Thus, counting of mismatched binding sites is not likely to give a significant improvement for the prediction of PCR success.

## Conclusion

We have created a novel method for masking repeated regions in sequences, detecting all PCR primer binding sites and possible PCR products from the human genome. The GENOMEMASKER package is suitable for researchers who need to evaluate or design unique PCR primers in genomic scale. It is able to mask the entire human genome for non-unique primers within 6 hours and find locations of all binding sites for 10 000 designed primer pairs within 10 minutes.

## Availability and requirements

Project name: GENOMEMASKER package

Project homepage: 

Operating system: Unix/Linux

Programming language: C/C++

Other requirements: None

License: The package is freely available to academic users

Any restrictions to use by non-academics: Licence needed

The web client for the GenomeTester program is available at  and the web client for the GenomeMasker is available at .

## Authors' contributions

RA conducted this study, carried out different tests on various methods, validated the package and was responsible for drafting the manuscript. ER created the initial code of the package and LK optimized the code. MR contributed to the conception of this study, participated in its design and coordination and helped to draft the manuscript. All authors have read and approved the final manuscript.

## Figures and Tables

**Table 1:** The effect of different repeat-masking methods onto PCR primer design.

	**PRIMER3 + No masking**	**PRIMER3 + DUST**	**PRIMER3 + Tandem** ** Repeats Finder**	**PRIMER3 + repeat library** ** (ALU+LINE+MIR)**	**PRIMER3 + Repeat Masker -qq**	**GM_PRIMER3 + Genome** ** Masker -wl 16**
**Primer selection** ** was not possible (no primers)**	2%	2%	2%	4%	31%	7%
**>10 predicted binding sites for** ** at least one primer of the primer pair**	52%	51%	51%	39%	12%	NA*

In this test, 1000 random regions, each 1000 bp long, were selected from the human genome for selection of PCR primers. The PRIMER3 program was used in combination with different masking methods to design PCR primers for amplification for each region. The binding sites were predicted for each designed primer using 16 nucleotides from the primer 3'-end and requiring perfect match with template DNA. *Masking the primers that have >10 predicted binding sites is an intrinsic feature of GenomeMasker and thus not comparable to others.
